# Vestigial-like 3 is a novel Ets1 interacting partner and regulates trigeminal nerve formation and cranial neural crest migration

**DOI:** 10.1242/bio.026153

**Published:** 2017-09-04

**Authors:** Emilie Simon, Nadine Thézé, Sandrine Fédou, Pierre Thiébaud, Corinne Faucheux

**Affiliations:** Univ. Bordeaux, INSERM U1035, F-33076 Bordeaux, France

**Keywords:** Vestigial-like, Ets1, *Xenopus*, Cranial neural crest, Trigeminal nerve, Wnt-FGF

## Abstract

*Drosophila* Vestigial is the founding member of a protein family containing a highly conserved domain, called Tondu, which mediates their interaction with members of the TEAD family of transcription factors (Scalloped in *Drosophila*). In *Drosophila*, the Vestigial/Scalloped complex controls wing development by regulating the expression of target genes through binding to MCAT sequences. In vertebrates, there are four *Vestigial-like* genes, the functions of which are still not well understood. Here, we describe the regulation and function of vestigial-like 3 (vgll3) during *Xenopus* early development. A combination of signals, including FGF8, Wnt8a, Hoxa2, Hoxb2 and retinoic acid, limits *vgll3* expression to hindbrain rhombomere 2. We show that vgll3 regulates trigeminal placode and nerve formation and is required for normal neural crest development by affecting their migration and adhesion properties. At the molecular level, vgll3 is a potent activator of *pax3*, *zic1*, *Wnt* and *FGF*, which are important for brain patterning and neural crest cell formation. Vgll3 interacts in the embryo with Tead proteins but unexpectedly with Ets1, with which it is able to stimulate a MCAT driven luciferase reporter gene. Our findings highlight a critical function for vgll3 in vertebrate early development.

## Abstract (precis)

**Summary:** Here, we provide the first description of Vgll3 function during vertebrate development. Vgll3 is a new partner of Ets1 and regulates trigeminal nerve formation and cranial neural crest cell migration.

## INTRODUCTION

The vestigial-like (VGLL) family of proteins takes its name from the *Drosophila* Vestigial (Vg), which is required for wing formation (Halder et al., 1998; Kim et al., 1996). Vestigial forms a co-transcriptional activator complex with the protein Scalloped (Sd), a member of the TEAD family of transcription factors, which activates genes involved in wing morphogenesis (Guss et al., 2001). Several *Vestigial-like* genes have been identified in vertebrates; all encode proteins with a Tondu domain that mediates interaction with TEADs (Bonnet et al., 2010; Chen et al., 2004; Faucheux et al., 2010; Maeda et al., 2002; Mielcarek et al., 2002, 2009; Simon et al., 2016).

Although the Vestigial function in *Drosophila* is well known, the roles played by vertebrate orthologs have not been fully explored to date. Mammalian VGLL2 is an essential cofactor of TEAD, able to stimulate muscle differentiation, and in zebrafish embryo it is involved in the development of the neural crest (NC) cell-derived craniofacial skeleton (Gunther et al., 2004; Johnson et al., 2011; Maeda et al., 2002). Mammalian VGLL4 acts, like its Drosophila homolog Tgi, as a repressor of the Hippo pathway (Chen et al., 2004; Guo et al., 2013; Koontz et al., 2013).

Vgll3 has received less attention, although the gene is the best conserved in the family in terms of structure and expression in the brain and nervous system (Simon et al., 2016). One peculiarity of vertebrate Vgll3 is the presence of a histidine repeat (six or more residues), a relatively uncommon feature with unknown function that is found in only 86 human proteins (Salichs et al., 2009). Several antagonist functions have been speculated for VGLL3 in human deduced from clinical observations. VGLL3 displays either a role in the tumor suppression pathway (Cody et al., 2009; Gambaro et al., 2013) or has oncogenic properties (Antonescu et al., 2011; Hallor et al., 2009; Helias-Rodzewicz et al., 2010). Very recently, VGLL3 has been identified as a regulator of a gene network that promotes female-biased autoimmunity (Liang et al., 2017).

We have described the expression pattern of the *vgll* family during *Xenopus* development, and shown that *vgll3* expression is tightly regulated in the embryo and restricted to rhombomere 2 (r2) of the hindbrain (Faucheux et al., 2010). We examine here the function of vgll3 during early development, and show that both gain and loss of vgll3 expression impairs trigeminal placode and nerve development and cranial neural crest (CNC) cell migration. We show that vgll3 can activate *pax3* and *zic1* expression not only in whole embryo but also in animal cap explants. In addition, vgll3 is able to activate Wnt and FGF signals, providing a model in which vgll3 acts via signaling molecules expressed in the hindbrain. Vgll3 can interact with tead1 and tead2 in the embryo, but this interaction is not sufficient to explain its properties suggesting other potential interacting proteins. We identified ets1 as a new partner of vgll3 that can account for *pax3* sustained expression in the embryo. Our results define vgll3 as an essential regulator of trigeminal nerve formation and CNC cell migration.

## RESULTS

### Restricted spatial expression of *vgll3* depends on multiple factors

**Fig. 1.**
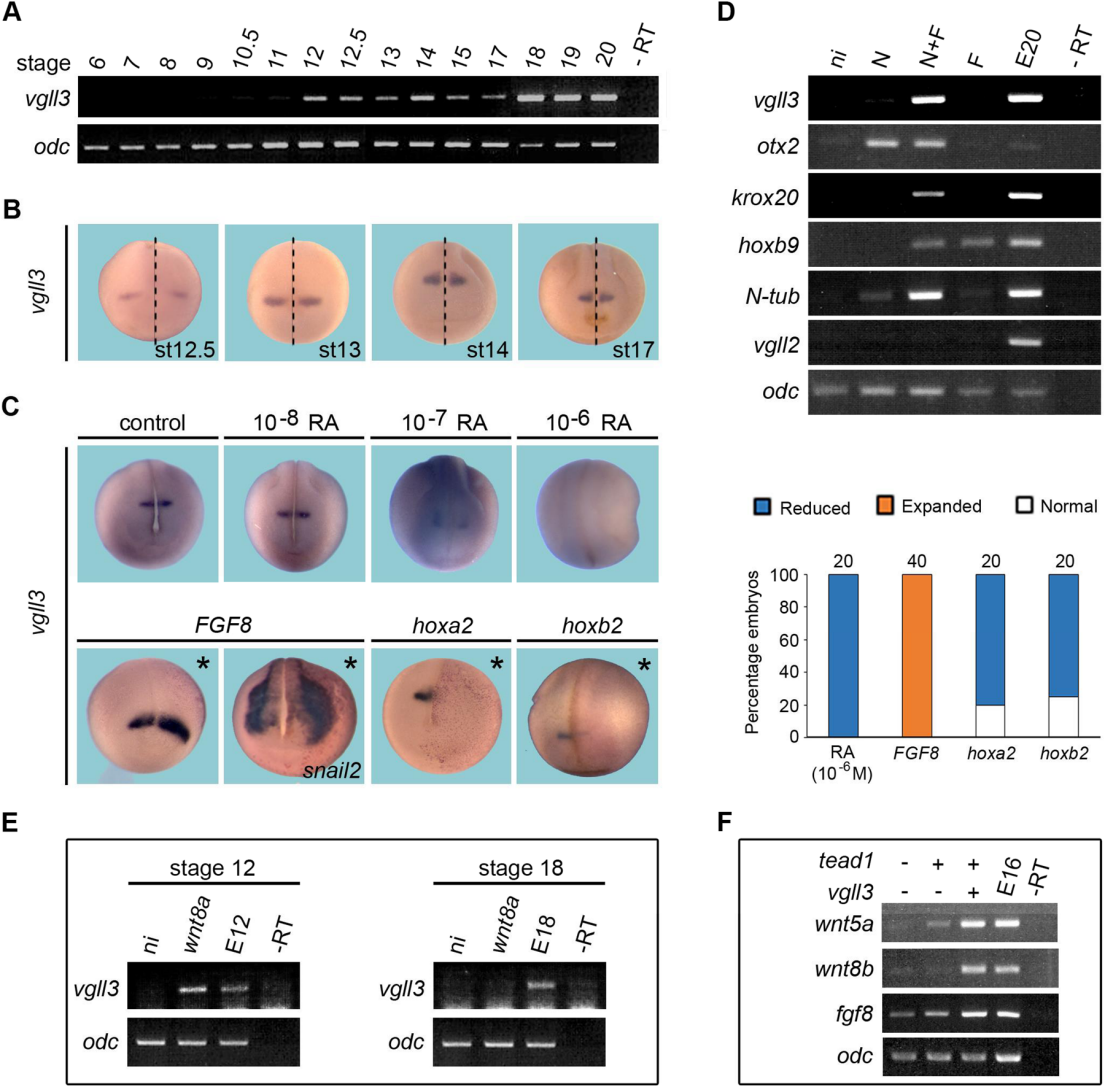
**Temporal expression and spatial regulation of *vgll3* in *Xenopus* embryo*.*** (A) *Vgll3* expression detected by RT-PCR starts between stages 11 and 12. (B) *Vgll3* is detected by ISH in r2 during neural tube closure. Dashed lines indicate the midline of embryos. (C) *Vgll3* expression decreases in stage 18 embryos treated with increasing concentrations of retinoic acid (RA). *FGF8* mRNA-injected embryos show an anterior-lateral enlargement of *vgll3* expression domain. *Hoxa2* or *hoxb2* mRNA-injected embryos show a strong reduction of *vgll3* expression. All views are dorsal-anterior. Asterisks indicate the injected side. Quantification of *vgll3* regulation results is shown in the right panel. Three independent experiments were performed. The number of embryos analyzed is indicated on the top of each bar. (D) *Vgll3* is induced in animal caps treated with noggin+FGF2 (N+F). (E) Vgll3 expression is induced in early, but not late, animal cap cells overexpressing wnt8a. (F) Overexpression of vgll3 in combination with tead1 in animal cap cells stimulates the expression of wnt5a, wnt8b and fgf8. E, noninjected embryo (number indicates the stage); ni, animal cap from uninjected embryo; N-tub, N-tubulin; –RT, no cDNA. *Ornithine decarboxylase* (*odc*) gene expression was used as a control.

To determine accurately the onset of *vgll3* expression after mid-blastula transition we performed reverse transcription polymerase chain reaction (RT-PCR) analysis on two-cell stage to stage 20 embryos with narrowing towards close stages between stages 10.5 and 15. *Vgll3* mRNA is detected in stage 12 embryos (Fig. 1A). Using whole-mount *in situ* hybridization (ISH), we detected *vgll3* in a single stripe across the neural plate in stage 12.5 (Fig. 1B). Between stage 13 and 17, the v*gll3* expression domain follows the neural tube closure as the space between the stripes on each side of the dorsal midline narrows. *Vgll3* staining decreases laterally but increases along the anterior-posterior axis. Therefore, *vgll3* is one of the earliest markers of the hindbrain and, to our knowledge, the only one for which expression is restricted to r2. Such a peculiarity makes it a good model for studying its regulation and function in relation to hindbrain patterning.

Hindbrain patterning depends on an intricate complex regulation network involving signaling pathways, such as those of fibroblast growth factor (FGF) and retinoic acid (RA), which establish a *Hox* code along the anterior-posterior axis (Alexander et al., 2009). Levels of RA vary along the anterior-posterior axis of the hindbrain, and *Xenopus* embryos treated with RA displayed loss of anterior hindbrain structures (Papalopulu et al., 1991). Similarly, embryos treated with RA showed a dose-dependent inhibition of *vgll3* expression (Fig. 1C). FGF8 mRNA-injected embryos also showed a lateral and anterior-lateral expansion of *vgll3* expression domain at the level of r2, with *snail2* expression used as control (Fig. 1C). This mimics the observations made on the effect of FGF8 overexpression on *krox20* expression (Fletcher et al., 2006).

We also used the animal cap assay to examine FGF-dependent regulation of *vgll3*. Neither FGF8 nor FGF2 induced *vgll3* expression (data not shown). Therefore, we tested *vgll3* expression in animal caps that were neuralized with the BMP inhibitor noggin. Noggin induces anterior neural fate cells, whereas FGF2 accounts for posterior neural induction (Delaune et al., 2005; Lamb and Harland, 1995). Animal caps from *noggin* mRNA-injected embryos or treated with FGF2 expressed the anterior marker *otx2* or the posterior marker *hoxb9*, respectively, but not *vgll3* nor *krox20* (Fig. 1D). Animal caps derived from *noggin* mRNA-injected embryos and treated with FGF2 expressed both *vgll3* and *krox20* (Fig. 1D). Neural induction is independent of mesoderm as controlled by the absence of *vgll2* muscle-specific expression (Fig. 1D).

We next determined whether *vgll3* expression could be regulated by *hox* genes. The anterior limits of *hoxa2* and *hoxb2* expression in the vertebrate hindbrain are r1/r2 and r2/r3 borders, respectively (Baltzinger et al., 2005; Moens and Prince, 2002; Nonchev et al., 1996; Schilling et al., 2001). When embryos were injected either with *hoxa2* or *hoxb2* mRNAs they showed reduced *vgll3* expression in r2 (Fig. 1C). We next studied the effects of secreted signaling Wnt proteins involved in many aspects of neural development (Baker et al., 1999). Wnt8 overexpression in animal cap cells stimulates vgll3 expression (Fig. 1E) and, conversely, vgll3 stimulates wnt8, and also wnt5a and fgf8 (Fig. 1F).

Together, these data suggest that *vgll3* expression in hindbrain is positively regulated by FGF and Wnt signals and negatively by *hox* genes and RA signal. Vgll3 can stimulate secreted molecule members of the canonical and noncanonical Wnt and FGF pathways.

### *Vgll3* regulates trigeminal placode and nerve formation

**Fig. 2.**
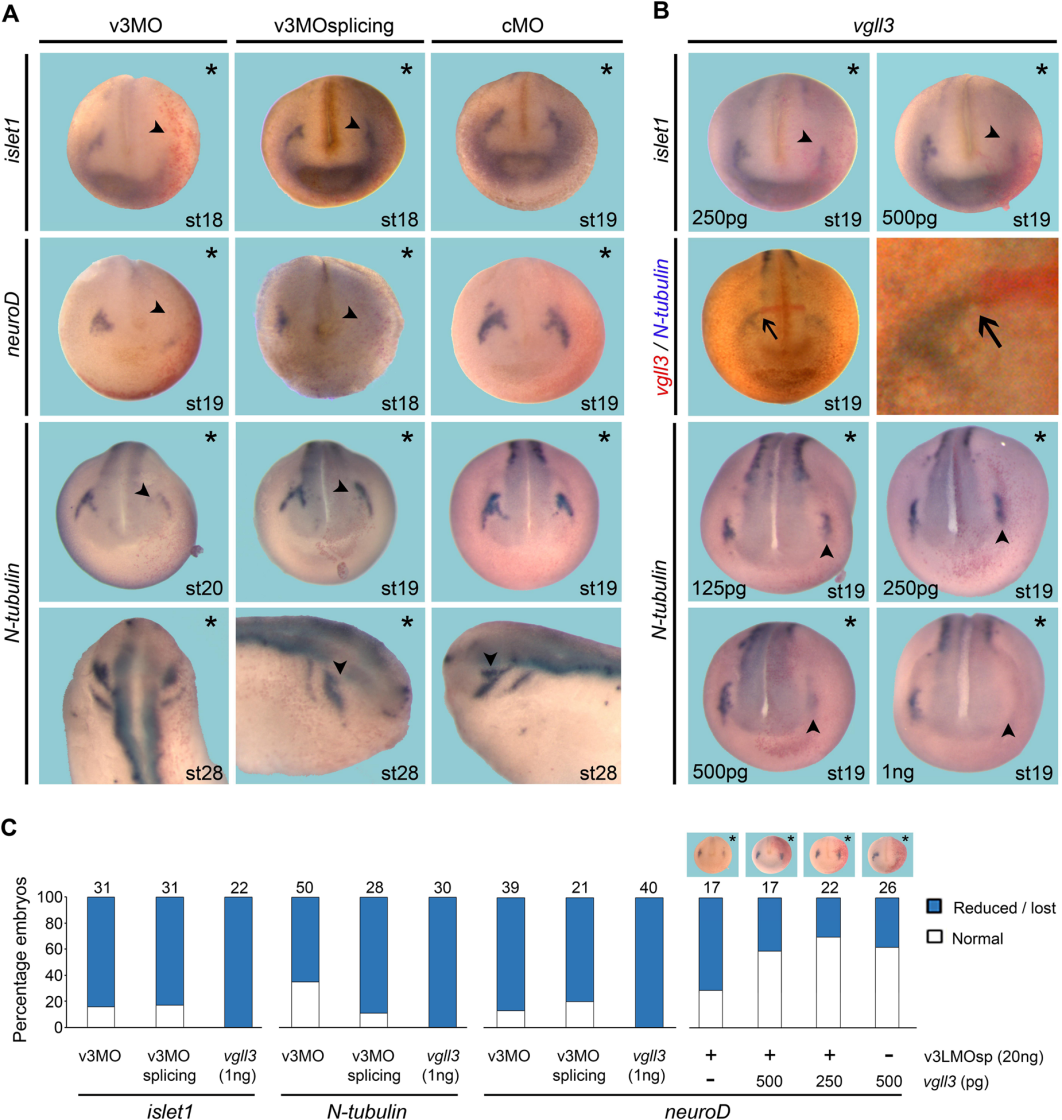
***Vgll3* knockdown or overexpression impairs trigeminal placode and nerve formation*.*** (A) Embryos injected with v3MO or v3MOsplicing (v3LMOsp and v3SMOsp, 20 ng each) exhibit reduced expression of *islet1*, *neuroD* and *N-tubulin* in the trigeminal placodes (arrowheads). (B) Overexpression of increasing amounts of *vgll3* mRNA reduces *islet1* and *N-tubulin* expression in stage 19 embryos. Double ISH shows no prominent overlapping staining between *vgll3* (red) and *N-tubulin* (blue) (arrow). The injected side (indicated with asterisks) was traced by *lacZ* staining. Gene expression was assayed by ISH. Arrowheads indicate the trigeminal placodes. (C) Quantification of results. Images at the top of bars indicate v3LMOsp defects rescued with increasing amounts of *vgll3* mRNA injections. Three independent experiments were performed. The number of embryos analysed is indicated on the top of each bar. Views are dorsal-anterior excepted for lateral views for stage 28 embryos.

Trigeminal ganglion that will give rise to trigeminal nerve has a dual embryonic origin being derived from both NC and epidermal placode (Hamburger, 1961; Steventon et al., 2014). Therefore, we investigated whether neurogenesis was altered in vgll3-depleted embryos using markers of early trigeminal placode and the postmitotic neuronal marker *N-tubulin*. In stage 14 embryos, *vgll3* expression does not colocalize with expression of the trigeminal placode genes *islet1*, *neuroD*, *pax3* and *foxi1c* (Jeong et al., 2014), while in stage 20 embryos, their expression domains become closer (Fig. S1). We next used a morpholino (MO) antisense (v3MO) that blocks *vgll3* mRNA translation (Fig. S2). An additional morpholino was designed to inhibit *vgll3.L* and *vgll3.S* splicing (v3MOsplicing), the efficiency of which was controlled by RT-PCR (Fig. S3). In morphant embryos injected with v3MO or v3MOsplicing, *islet1*, *neuroD* and *N-tubulin* expression was partially or totally inhibited in prospective trigeminal and profundal placodes (arrowhead, Fig. 2A). This effect is dose-dependent (data not shown) and, in stage 28 embryos, the ophthalmic branch of the trigeminal nerve is shortened (50%, *n*=20, arrowhead, Fig. 2A). This effect is specific since the vgll3.L splicing morphants can be rescued with the injection of *vgll3* mRNA (Fig. 2C). Of note, a stronger effect was observed when both v3MO splicing were co-injected (Fig. S4). The function of *vgll3* on the trigeminal formation was confirmed at later stages (Fig. S5) and by using a second translational MO (Fig. S6).

Stage 19 embryos overexpressing *vgll3* mRNA showed a dose-dependent decrease of *islet1* and *N-tubulin* expression at the level of trigeminal placodes (arrowhead, Fig. 2B). The effects observed did not result from apoptosis as controlled by TUNEL labeling (Fig. S5). Together, these results indicate that trigeminal placode and nerve formation requires a strictly controlled *vgll3* expression level.

### Knockdown of vgll3 does not affect CNC formation but causes defects in their derivatives

**Fig. 3.**
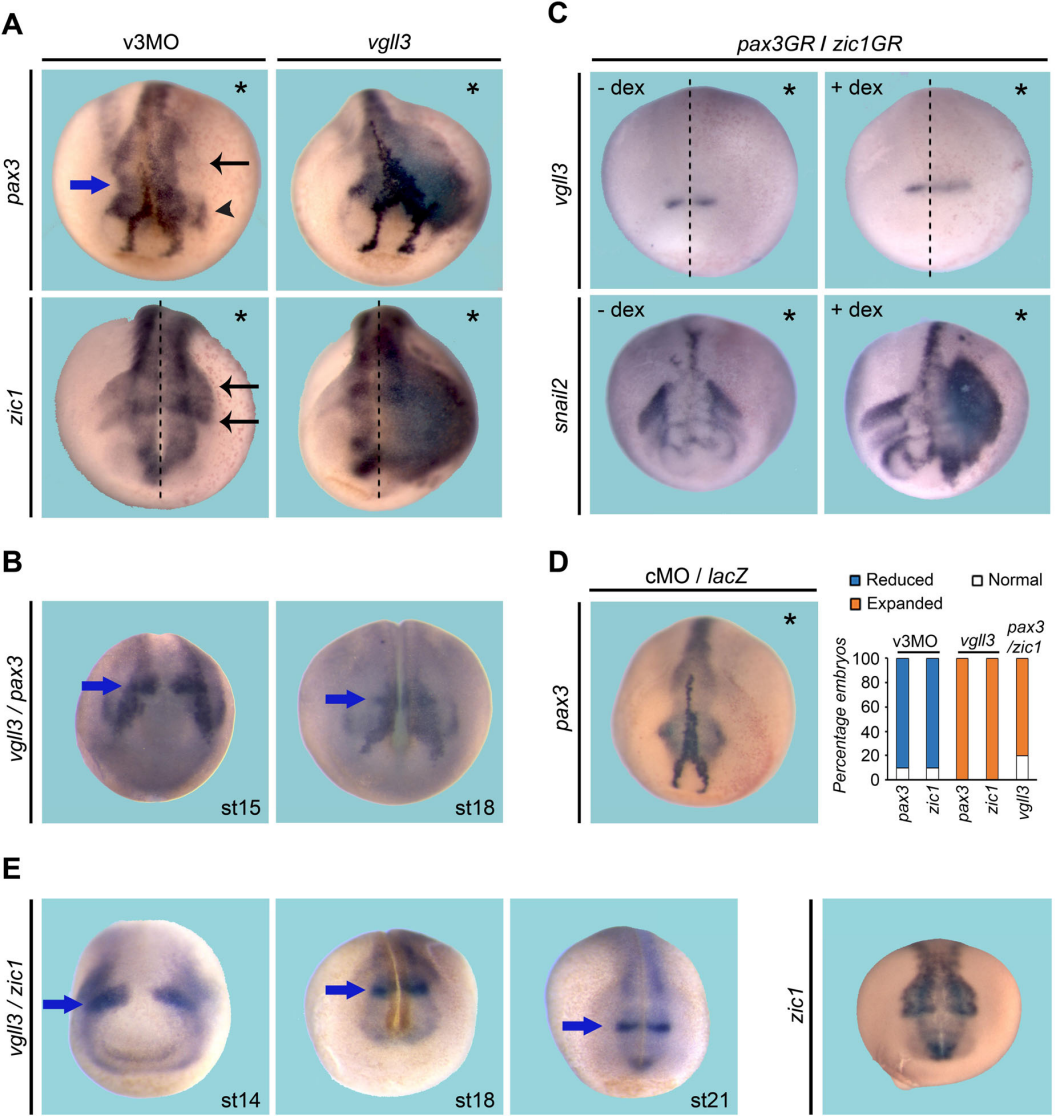
***Vgll3* stimulates *pax3* and *zic1* expression.** (A) Embryos were injected with v3MO or *vgll3* mRNA and analysed at stage 19 for *pax3* and *zic1* expression. Defects of CNC lateral streams and trigeminal placode are shown by black arrows and arrowhead, respectively. (B) ISH for *vgll3* and *pax3* shows overlapping expression at the r2 level (blue arrows indicate *vgll3* expression domain). (C) Stage 19 embryos injected with *pax3GR/zic1GR* mRNAs show a faint *vgll3* and a strong *snail2* expression after dexamethasone treatment (+dex). (D) cMO/LacZ mRNA control and quantification. Three independent experiments were performed (*n*=40). Asterisks indicate the injected side. All views are dorsal-anterior. Dashed lines indicate the midline of embryos. (E) ISH for *vgll3* and *zic1* shows overlapping expression at the r2 level (blue arrows). Zic1 expression in stage 21 embryo.

All rhombomeres produce CNC cells and those originating from r2 will populate pharyngeal arch 1 in coordination with CNC cells from r1 and r3 (Lumsden et al., 1991; Sechrist et al., 1993). In the genetic regulatory network, *pax3* and z*ic1* have been shown to be essential for specification, differentiation and migration of CNC cells in *Xenopus* (Bae et al., 2014; Betancur et al., 2010; Milet et al., 2013). Stage 19 embryos depleted for vgll3 showed a decrease in *pax3* and *zic1* (Fig. 3A). In those embryos, the lateral streams of CNC cells have either disappeared or have fused (black arrows, Fig. 3A). This is in agreement with the partial colocalization of *vgll3* with *pax3* and *zic1* expression (Fig. 3B,E). Vgll3 depletion affected pax3-profundal placode formation, as previously shown (arrowhead, Fig. 3A).

**Fig. 4.**
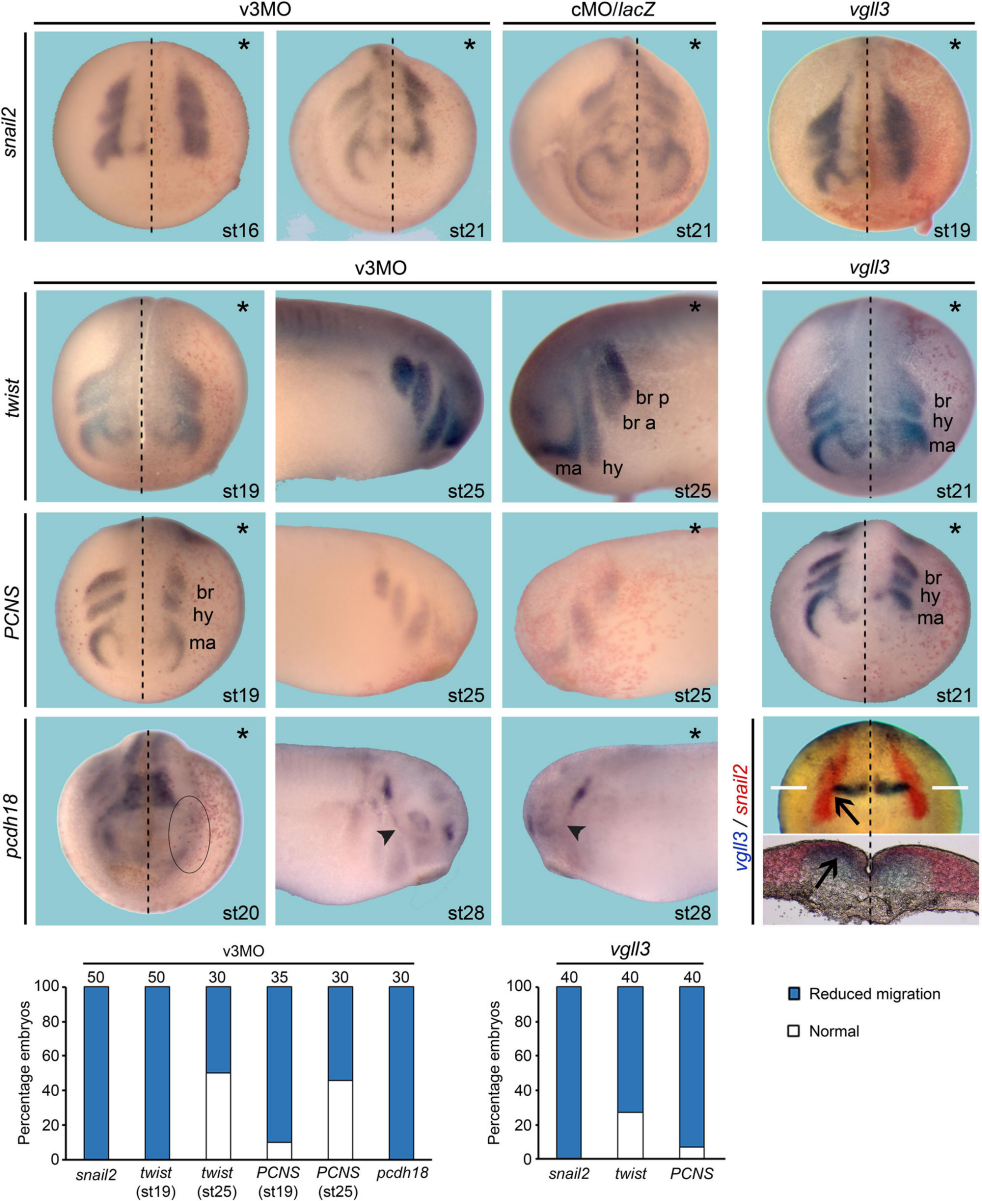
***Vgll3* knockdown and overexpression do not affect CNC formation but block their migration.** Embryos were injected with v3MO (40 ng or cMO) or *vgll3* mRNA (1 ng, or *lacZ* mRNA) and analysed at different stages for *snail2*, *twist*, *PCNS* or *pcdh18* expression. Pharyngeal arches are indicated (a, anterior; br, branchial; hy, hyoid; ma, mandibular; p, posterior). Arrowheads indicate the mandibular branch of the trigeminal nerve. Vgll3 knockdown and overexpression block migration of CNC streams. Arrows indicate overlapping expression of *vgll3* and *snail2*. White lines indicate the plane of agarose section. Asterisks indicate the injected side. Dashed lines indicate the midline of embryos. The oval indicates the lateral CNC stream. All views are dorsal-anterior except lateral views for stage 25 and 28 embryos. Quantification of results is shown in the lower panels. Three independent experiments were performed. The number of embryos analysed is indicated at the top of each bar.

*Vgll3* mRNA-injected embryos showed strong ectopic expression of *pax3* and z*ic1*, while no change was observed in embryos injected with cMO or *lacZ* mRNA (Fig. 3A,D). Because *pax3* and *zic1* are expressed earlier than *vgll3* in the developing embryo we examined whether they could regulate its expression (Hong and Saint-Jeannet, 2007). Embryos injected with inducible *pax3GR/zic1GR* mRNAs showed a faint focalized lateral expansion of *vgll3* expression after dexamethasone treatment (Fig. 3C). Together, these data suggest that *vgll3* regulates *pax3* and *zic1* expression and can be stimulated, albeit very faintly, by pax3 and zic1. *Snail2* is one of the earliest CNC specifiers expressed in the embryo followed by *twist* (Lander et al., 2013; Mayor et al., 1995). Whole-mount ISH for *vgll3/snail2* shows a lateral and partial overlapping expression at the r2 level (arrows, Fig. 4). In stage 16 vgll3-depleted embryos, expression of *snail2* is not affected, but the onset of CNC migration is blocked, and this is more conspicuous in stage 21 embryo (Fig. 4). Stage 19 and stage 25 vgll3-depleted embryos display a reduction of *twist* expression in mandibular, hyoid and branchial segments (Fig. 4).

CNC migration is regulated by cell-cell interaction mediated by cadherins such as PCNS (protocadherin in NC and somites) and pcdh18 (Aamar and Dawid, 2008; Rangarajan et al., 2006). In stage 19 vgll3-depleted embryos, *PCNS* expression is less extended along the different streams that will form pharyngeal arches and the embryos showed a loss of *PCNS* expression in stage 25 (Fig. 4). Likewise, *pcdh18* expression is not detected in the CNC lateral streams in stage 20 morphant embryos (circle, Fig. 4) and absent in the mandibular branch of trigeminal nerve in stage 28 (arrowhead, Fig. 4). Similar results were obtained with a second translational morpholino (Fig. S6) and in v3MOsplicing morphants (data not shown).

Stage 19 and stage 21 embryos overexpressing *vgll3* mRNA showed a clear impairment of cell migration expressing *snail2* and *twist* (Fig. 4). CNC cell migration into pharyngeal arches is also inhibited in vgll3-overexpressing embryos as revealed by *PCNS* staining (Fig. 4). Taken together, these results suggest that the absence of vgll3 does not affect CNC formation but impairs their migration. Because CNC are the source of most of the cranial cartilages and play an important role in determining the head shape, we further observed that vgll3 depletion or overexpression induced abnormal cartilage and impaired head structures (Fig. S7).

### Vgll3 regulates CNC migration

**Fig. 5.**
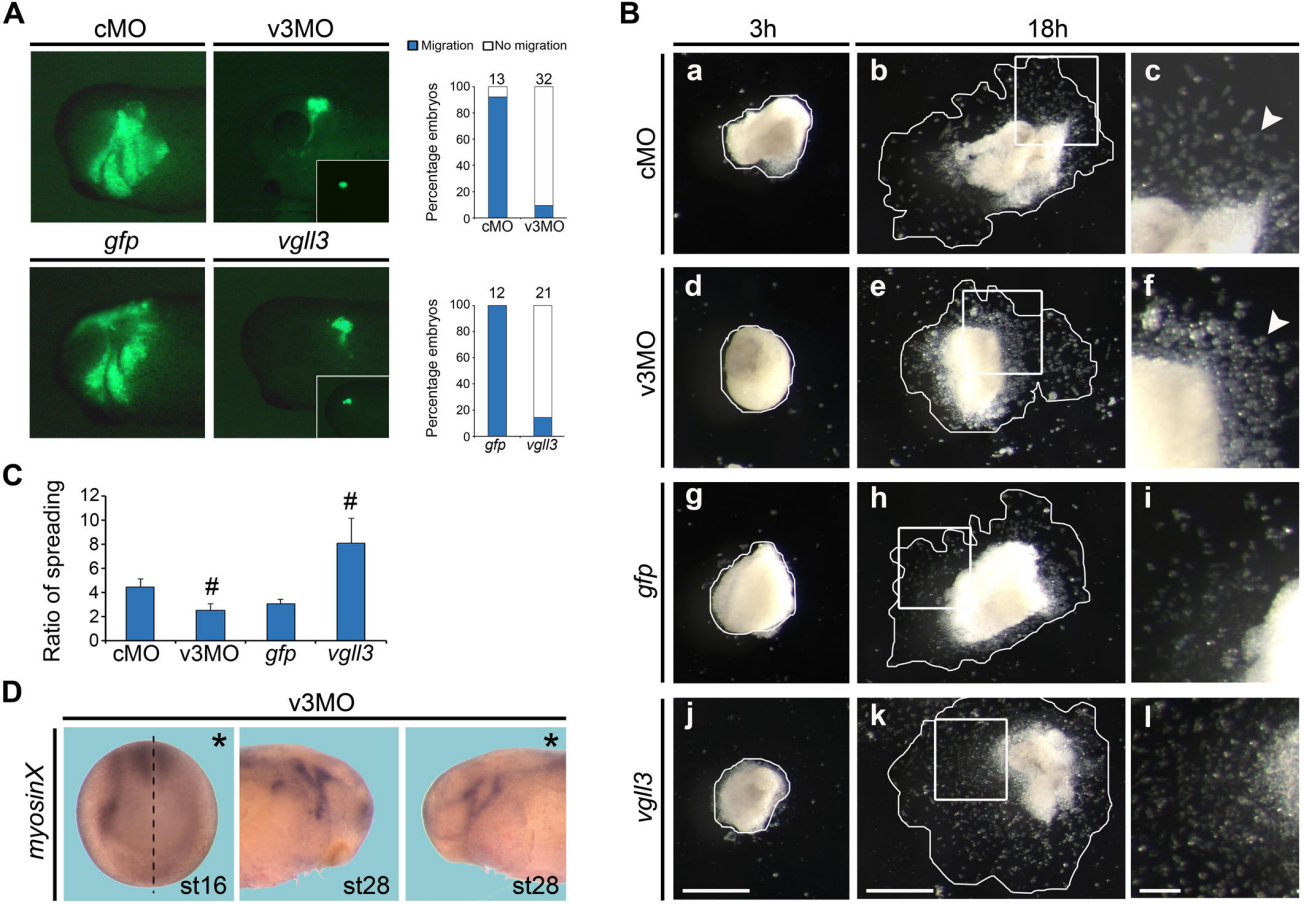
***Vgll3* regulates CNC migration*.*** CNC from neurula embryos injected with tracer *gfp* mRNA and v3MO (40 ng or cMO) or *vgll3* mRNA (1 ng) were (A) grafted on wild-type embryos at stage 17 and migratory phenotype was analysed by GFP fluorescence 18 h after transplantation (quantification of results in the right panel; insets show GFP-positive grafted cells just after transplantation; magnification has been adjusted to reduce the size of original images) or (B) plated on fibronectin and analysed 3 h (a,d,g,j) and 18 h (b,e,h,k) after plating. Enlarged views of the boxed areas are also shown (c,f,i,l). Scale bars: 1 mm (j,k); 250 µm (l). (C) Ratio of spreading measured by comparing the relative surface area between 18 h and 3 h of culture (indicated by the outlined areas in a,b,d,e,g,h,j,k). ^#^*P*<0.05; Student's *t*-test; data are mean±s.e.m. (D) *MyosinX* expression in stage 16 (dorsal-anterior view) or stage 28 (lateral view) embryos. Asterisks indicate the injected side. Dashed line indicates the midline of the embryo.

To investigate the implication of vgll3 in CNC migration, we performed transplantation experiments with green fluorescent protein (GFP) as a lineage tracer (Borchers et al., 2000). CNC from v3MO- or *vgll3*-mRNA injected embryos showed an inhibition of cell migration (Fig. 5A). To further analyze the role of vgll3 in cell migratory behavior, CNC explants were cultured on fibronectin-coated plates (Alfandari et al., 2003). At 3 h after plating, cells started to spread on their substrate (Fig. 5Ba,d,g,j). After 18 h, CNC explants from vgll3-depleted embryos displayed a reduced spreading compared to cMO CNC (Fig. 5B, e versus b). In contrast, explants from *vgll3* mRNA-injected embryos showed an enhanced spreading (Fig. 5B, k versus h). At higher magnification (Fig. 5Bc,f,i,l), only CNC cells from *vgll3*-depleted embryos seemed to show a spreading failure; instead, cells have tendency to dissociate from each other and remain round (Fig. 5B, f versus c, arrowheads). No apoptotic process was detected at this stage in morphant embryos (Fig. S5). Quantification analysis indicates that explants from *vgll3*-depleted embryos spread 1.8 less than cMO explants, while *vgll3* mRNA injected explants spread 2.6 more than control *gfp* explants (Fig. 5C). Embryos depleted for vgll3 showed a reduction of *myosinX* expression, known to be critical for cell-cell adhesion (Nie et al., 2009) at premigratory (stage 16) and migratory stages (stage 28), respectively (Fig. 5D). These findings suggest that *vgll3* is required for proper CNC cell migration through alteration in their spreading and adhesion properties.

### *Vgll3* regulates a specific subset of genes and interacts with tead in the embryo

**Fig. 6.**
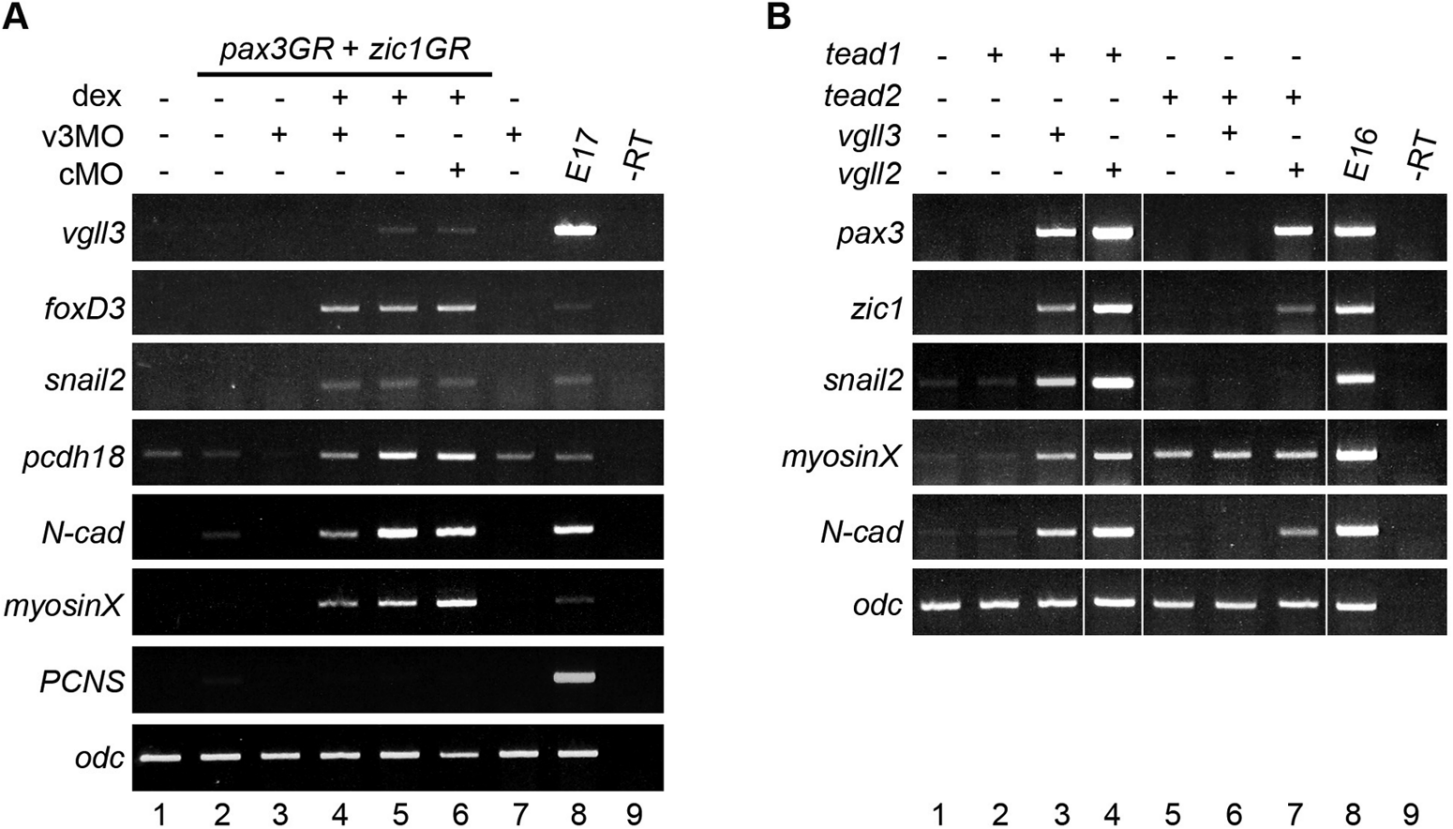
***Vgll3* regulates a specific subset of genes in animal cap explants.** (A) Embryos were injected with *pax3GR/zic1GR* and v3MO (40 ng or cMO), and treated with or without dexamethasone (±dex) before animal caps dissection and analysis by RT-PCR. (B) Embryos were injected with *myc*-*tead1* (T1), *myc*-*tead2* (T2), *HA*-*vgll3* (V3) or *HA*-*vgll2* (V2) mRNAs and animal caps were dissected and analysed by RT-PCR. Vertical white lines indicate spliced images in order to remove results not described in this paper. E16/E17, control stage 16/17 embryos; –RT, no cDNA.

We turned to the animal cap assay to gain further insight into the regulatory interplay between *vgll3*, *pax3* and *zic1* (Fig. 6A). Animal caps from embryos injected with *pax3GR* and *zic1GR* mRNAs in combination or not with v3MO expressed the CNC markers *foxD3* and *snail2* (lanes 4-5). This indicates that the activation of *foxD3/snail2* downstream of *p*ax3/*zic1* is independent of vgll3. However, *pcdh18*, *N-cadherin* (*N-cad*) and *myosinX* expression is significantly reduced in the presence of v3MO (compare lane 4 to lane 5 in Fig. 6A). In all experiments, no significant effect was observed in cMO injections (lane 6). We may conclude that although vgll3 is not essential for CNC induction, it is required for the full expression of genes involved in adhesion and migration of CNC downstream of pax3/zic1.

We next tested the effect of vgll3 overexpression on gene targets in combination with tead (Naye et al., 2007). None of the genes tested is activated by vgll3, tead1 or tead2 alone, excepted for myosinX that is induced by tead2 (data not shown and Fig. 6B, lanes 2 and 5). However, *pax3*, *zic1*, *snail2*, *myosinX* and *N-cadherin* are robustly expressed when *vgll3* is co-expressed with *tead1* (lanes 2 and 3). The co-expression of *vgll2* with *tead1* gave the same results (lane 4). Surprisingly, co-expression of *vgll3* and *tead2* did not stimulate any of the genes analyzed while *vgll2* and *tead2* did, albeit at different levels (lanes 6 and 7). Together, these results indicate that vgll3/tead1 can stimulate the expression of members of the gene regulatory network that orchestrate CNC formation and development.

**Fig. 7.**
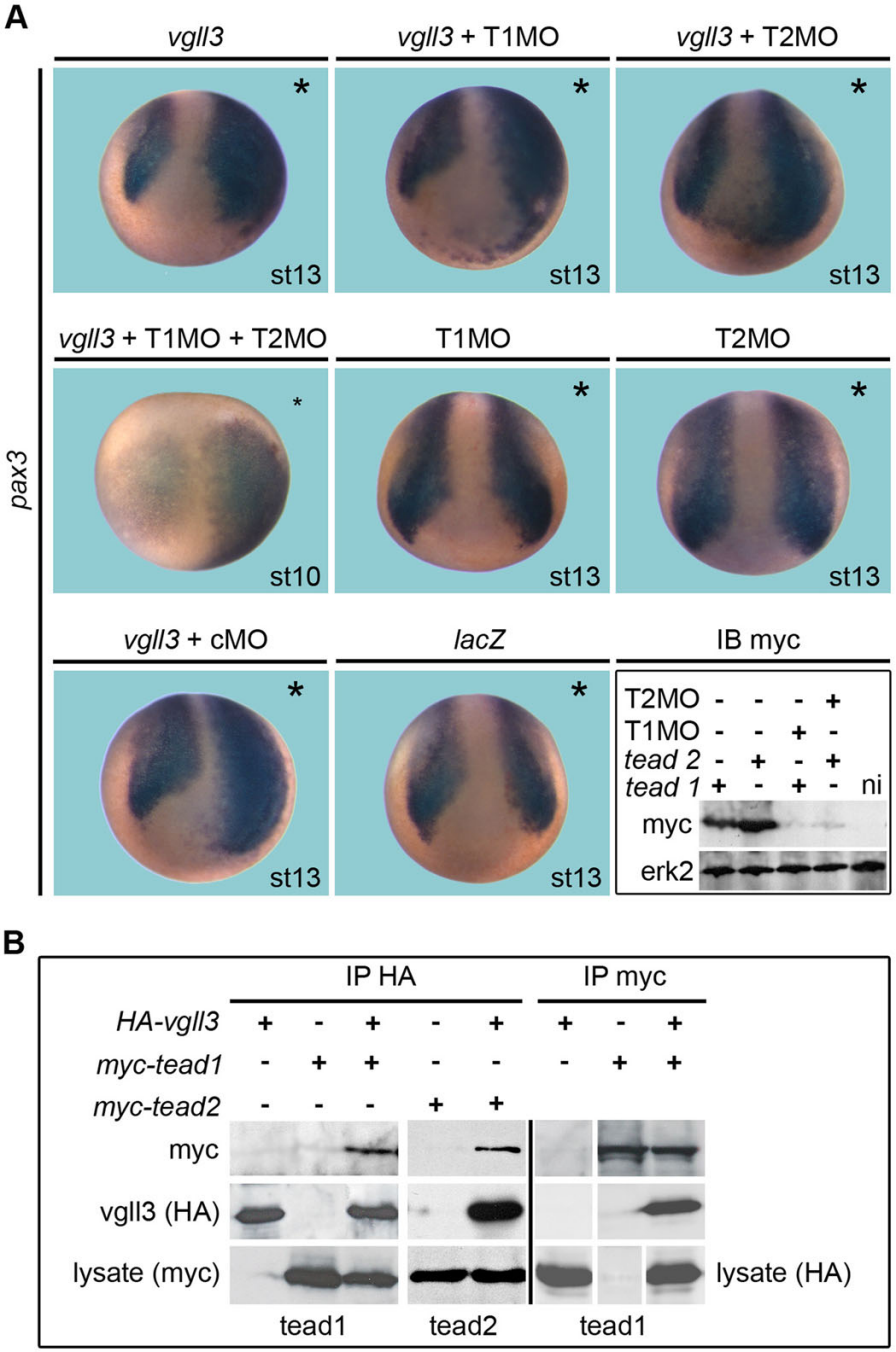
**Vgll3 interacts with tead proteins and can activate *pax3* independently of tead.** (A) Embryos injected with *vgll3* (1 ng or *lacZ*) mRNA and T1MO/T2MO or cMO were fixed at stage 10 or 13 and analysed for *pax3*. Vgll3 induces ectopic *pax3* expression when tead1 and tead2 have been knocked down. Lower right panel (IB myc): embryos were injected with 50 pg *myc-tead1* or *myc-tead2* mRNAs with 15 ng T1MO or T2MO and analysed by immunoblotting (IB). T1MO and T2MO efficiently block tead1 and tead2 expression, respectively. Erk2 was used as a control. ni, noninjected embryo. (B) Embryos injected with *HA*-*vgll3*, *myc-tead1* or *myc-tead2* mRNAs were processed for immunoprecipitation with HA (IP HA) or myc antibodies (IP myc) followed by IB with antibodies. Vgll3 interacts with tead1 and tead2 in the embryo. Lysate, IB of injected embryo before immunoprecipitation. Vertical white lines indicate spliced images in order to remove results not described in this paper (IP myc).

Tead1 has been previously shown to expand *pax3*-expressing CNC progenitors in *Xenopus* embryos and Tead2 has been found to be an endogenous activator of *Pax3* in mouse NC cells (Gee et al., 2011; Milewski et al., 2004). Therefore, we asked whether vgll3-dependent stimulation of *pax3* required tead1 or tead2. Embryos injected with *vgll3* mRNA and depleted for tead1, tead2 or both showed an extended *pax3* expression domain similar to embryos overexpressing vgll3 alone or injected with cMO (100%, *n*=50, Fig. 7A). We next demonstrated by immunoprecipitation that vgll3 could interact efficiently with tead1/tead2 (Fig. 7B). The above finding led us to hypothesize that even in the absence of tead1 and tead2, vgll3 is still able to activate *pax3* expression through a tead-independent mechanism.

### Vgll3 interacts with ets1 and requires a highly conserved histidine repeat to activate *pax3*

**Fig. 8.**
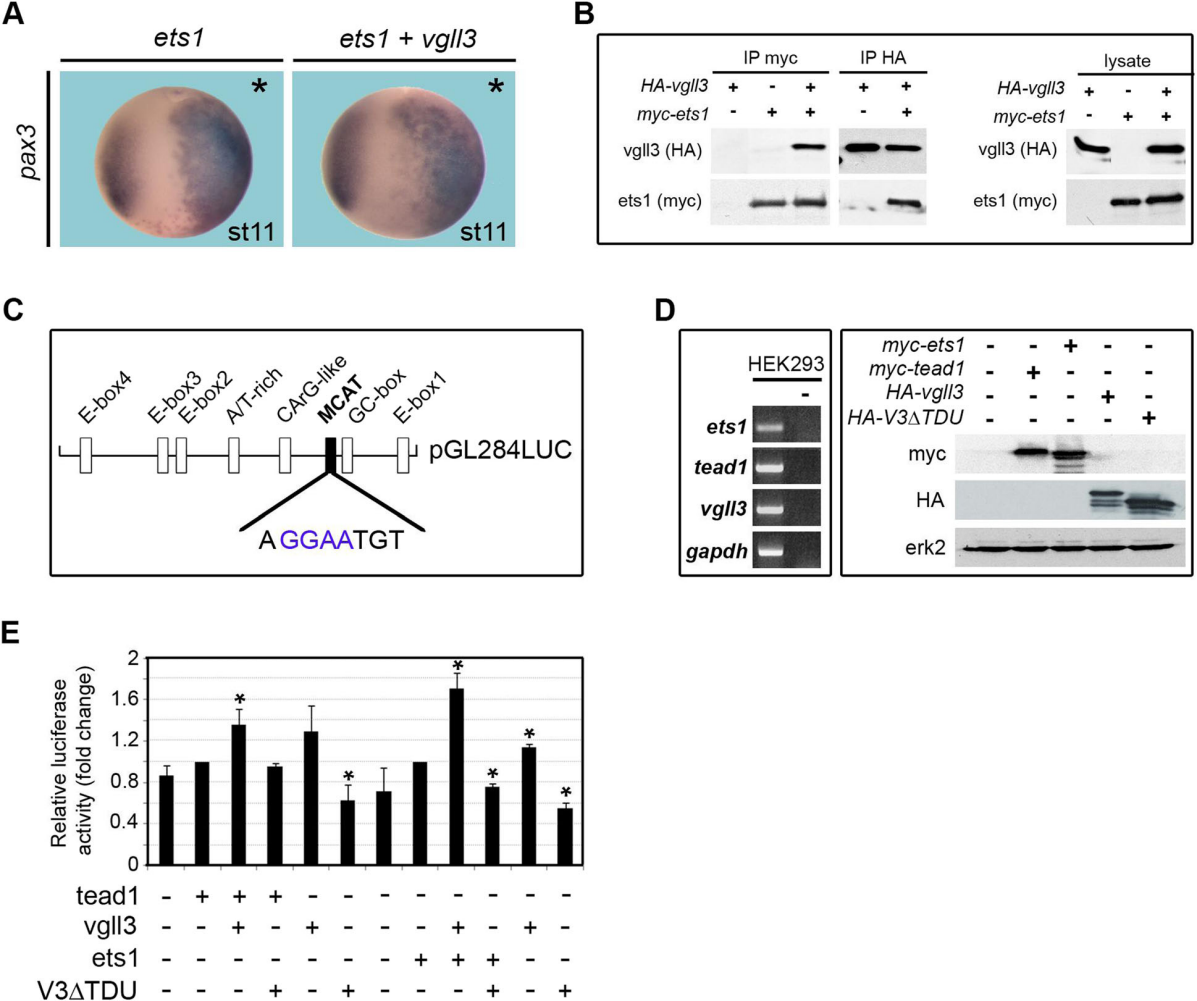
**Vgll3 interacts with ets1 and activates a tead-dependent luciferase reporter gene.** (A) *Pax3* expression in embryos injected with *vgll3* or *ets1* mRNA. Ets1 overexpression induces ectopic *pax3* expression as well as co-expression of ets1 and vgll3*.* Asterisks indicate the injected side. (B) Embryos were injected with *myc-*ets1 or *HA-vgll3* mRNAs. Immunoprecipitation with myc (IP myc) or HA antibodies (IP HA) was followed by IB with antibodies. (C) Schematic representation of the pGL284LUC reporter plasmid used in transfection assay. The different *cis*-sequences are depicted together with the MCAT *cis*-sequence (black box). (D) Left panel: e*ts1*, *tead1 and vgll3* are expressed in HEK293 cells when analysed by RT-PCR. (−), no cDNA. Right panel: HEK293 cells were transfected with *HA-vgll3*, *HA-V3ΔTDU*, *myc-tead1* or *myc*-*ets1* plasmids and similar amounts of proteins were checked by IB with myc and HA antibodies. *Gapdh* and erk2 are used as control. (E) HEK293 cells were co-transfected with pGL284LUC and *HA-vgll3*, *HA-V3ΔTDU*, *myc-tead1* and *myc-ets1* plasmids. Relative luciferase activity is expressed as fold change in luciferase activity compared to tead1 or ets1 alone. Data are mean±s.e.m. from three independent experiments carried out in duplicate. *P<0.05; Student's *t*-test.

Tead transcription factors bind the so-called MCAT sequence [5′-(AGGAATGT)-3′] present in non-muscle and muscle genes (Pasquet et al., 2006; Yoshida, 2008). For instance, tead binding sites have been identified in *Xenopus* and mouse *pax3* gene regulatory regions (Gee et al., 2011; Milewski et al., 2004). Surprisingly, the core sequence of TEAD binding site, 5′-GGAA-3′, is a perfect recognition sequence for members of the ETS domain transcription factor family (Sharrocks, 2001). Ets1, the prototype of the ETS family, is specifically expressed by CNC in the chick embryo and is necessary for their proper delamination (Théveneau et al., 2007). In *Xenopus*, *ets1* is expressed in neural tube and CNC and has been shown to be an immediate-early target gene of *pax3* (Meyer et al., 1997; Plouhinec et al., 2014). Indeed, embryos overexpressing ets1 showed an ectopic *pax3* expression (100%, *n*=30, Fig. 8A). A synergic effect of both ets1 and vgll3 on *pax3* expression is barely detectable owing to their strong effect when proteins are expressed alone (Fig. 8A). However, immunoprecipitation revealed that vgll3 could interact with ets1 in the embryo (Fig. 8B). To address the functionality of vgll3/ets1 complex, we turned to a gene reporter analysis. We have previously shown that a 284 bp sequence of the *α-tropomyosin* gene contained a MCAT binding site that could recapitulate endogenous gene expression pattern in a tead1-dependent way (Fig. 8C) (Pasquet et al., 2006). A luciferase reporter gene driven by this 284 bp fragment (pGL284LUC) was co-transfected in HEK293 cells with plasmids encoding HA-vgll3, myc-ets1 or myc-tead1. In those experiments, *Ets1*, *vgll3* and *tead1* are expressed at basal level in nontransfected cells and expressed at similar protein levels in transfected cells (Fig. 8D). Tead1 overexpressing cells showed a basal luciferase activity that is stimulated 1.35-fold upon co-expression of vgll3 (Fig. 8E), while luciferase activity of ets1-overexpressing cells is stimulated 1.7-fold. This difference might reflect a preferential activation of the reporter gene in favor of vgll3/ets1 rather than vgll3/tead1. Vgll proteins interact physically and functionally with TEAD proteins though their conserved tondu (TDU) domain (Vaudin et al., 1999). Vgll3 protein deleted from its TDU domain (V3ΔTDU) did not stimulate the luciferase activity in the presence of tead1 or ets1 (Fig. 8E). These results demonstrate that vgll3 can interact with ets1 and stimulate a MCAT element-dependent gene promoter. Moreover, the TDU domain of vgll3 is necessary for both ets1, and tead1-dependent gene activation.

All vertebrate Vgll3 proteins have in common a histidine tract, a feature that is shared by a limited number of proteins in mammals, the function of which is still speculative (Fig. S8A) (Salichs et al., 2009). When the protein is deleted from its histidine repeat (vgll3Δhis), it cannot stimulate anymore *pax3* expression (Fig. S8B) while its nuclear localization is unchanged (white arrow, Fig. S8C). In conclusion, the histidine repeat of vgll3 is required for its transcriptional activity but does not influence its nuclear localization.

## DISCUSSION

In the present study, we described vestigial-like 3 (vgll3) as a novel factor that has a dual role in trigeminal placode and nerve formation and NC migration. We identified vgll3 as a new cofactor of ets1 that can regulate, through its association, MCAT-dependent gene expression. Finally, we have provided evidence that the histidine-rich repeat, which is a unique feature to vgll3 proteins, is essential for its activity.

### Vgll3 expression is strictly restricted to rhombomere 2 and regulates trigeminal placode and nerve formation

We showed that *vgll3* expression is spatially restricted in the hindbrain through a combination of multiple signals including retinoic acid (RA), FGF8, Wnt, hoxa2 and hoxb2. This is consistent with previous findings that showed that FGF8 restricts the caudal boundary of anterior neural gene and our observation where engrailed2 overexpression switched off *vgll3* (Faucheux et al., 2010; Fletcher et al., 2006). We found that Vgll3 expression is caudally restricted by *hoxb2*. Surprisingly, hoxa2 overexpression also switches off *vgll3* expression, suggesting that *vgll3* is not subject to this repression in the normal development or is counteracted by positive signals. Both gain- and loss-of-function of hoxa2 in *Xenopus* embryos phenocopies our results on vgll3. Indeed, in both cases, embryos displayed skeletal head defects and NC cell migration impairment (Baltzinger et al., 2005; Pasqualetti et al., 2000). This fits with the hypothesis that hoxa2 could be a repressor of *vgll3* in r2.

Vgll3 gain- and loss-of-function clearly affected the expression of the specific placode genes *islet1* and *neuroD*. Consequently, *N-tubulin* expression is affected leading to a reduction in ophthalmic and maxillo-mandibular branches and in axonal outgrowth of trigeminal nerve. We hypothesize that *vgll3* regulates trigeminal placode development through *pax3 and zic1*, two genes that are associated with placode development (Jaurena et al., 2015; Schlosser, 2006). Vgll3-depleted embryos show a downregulation of *pax3* at the level of trigeminal placode, while *vgll3* overexpression induces *pax3* and *zic1* ectopic expression. In pluripotent animal cap cells, vgll3 overexpression also stimulates *pax3* and *zic1* expression. Surprisingly, *vgll3* is not expressed in placode domain and therefore we may suggest that it acts in a non-cell autonomous manner. Indeed, it is known that Wnt and FGF signals cooperate in the formation and differentiation of the otic and trigeminal placodes (Canning et al., 2008; Park and Saint-Jeannet, 2008). Since we have showed that vgll3 stimulates both Wnt and FGF expression, we hypothesize that vgll3 regulates trigeminal placode and nerve formation through these signals.

That similar phenotypes in *vgll3* gain- or loss-of-function studies are observed may be conceivable if we consider a functional dependence on protein-protein interaction where proper stoichiometry is essential (Lander et al., 2013). In our case, this could be related to the formation of the complex between vgll3 and tead1 (or ets1) and several mechanisms of repression can be proposed such as competition, quenching or squelching of the transcriptional complex.

### *Vgll3* is implicated in signaling pathways that control migration of CNC cells

Although vgll3 is a strong activator of *pax3* and *zic1*, its temporal expression precludes any role in the early NC gene regulatory network. However, from *in vivo* and *in vitro* analysis of morphant embryos, we may conclude that vgll3 is required for normal CNC migration as shown by the analysis of *snail2*-positive cells that do not migrate. How can we reconcile the broad effect of *vgll3* knockdown that affect all segments of the migrating CNC and their derivatives, while its expression is restricted to r2? We propose that *vgll3* can act on target genes through secreted molecules. Indeed, we have showed that vgll3 stimulates *wnt5a*, *wnt8b* and *fgf8*, supporting the hypothesis of a nonautonomous role through those signals. Moreover, this ensures the maintenance of *pax3* and *zic1* expression levels.

It is interesting to note that in zebrafish, vgll2a, a paralog of vgll3, has been shown to regulate CNC derivatives formation in a nonautonomous manner (Johnson et al., 2011). A recent report demonstrates that both activation and inhibition of canonical Wnt signaling results in severe NC migration in *Xenopus* embryo (Maj et al., 2016). This may explain our results since we have shown that vgll3 stimulates Wnt expression supporting a role through secreted molecules. We may also hypothesize a paracrine action like the one observed for en2 and pax2/5 that regulates wnt-1 and its target Tcf-4 in a nonautonomous manner during brain patterning (Koenig et al., 2010). *Vgll3* can also regulate cell fate in the hindbrain in a non-cell autonomous manner, as has been shown for *meis3* (Dibner et al., 2001).

The migration default of CNC induced by *vgll3* depletion can be correlated to myosinX which is required for adhesion of CNC cells to the extracellular matrix (Nie et al., 2009). Interestingly, *myosinX* and *vgll3* knockdown affect migration (this study and Grenier et al., 2009). Moreover, *vgll3* knockdown in embryos and in animal cap cells induced a specific decrease in *myosinX* expression, which may explain the inhibition of CNC cell migration *in vivo*. Together, our data establish a potential link between vgll3 and the myosinX-dependent migration processes (Nie et al., 2009; Zhu et al., 2007). After induction, CNC cells leave their original territory followed by a cadherin-dependent migration process (Théveneau and Mayor, 2012). *Vgll3* downregulation decreases *N-cadherin* and *pcdh18* expression in animal cap explants and *PCNS* and *pcdh18* expression in the embryo. Interestingly, vgll3-depleted embryos phenocopied twist1-depleted embryos leading to abnormal cartilage development (Lander et al., 2013). Surprisingly, a potential involvement of vgll3 in NC cells emerged from the report on a human patient that presents a microdeletion of chromosomal region 3p11.2-p12.1, including the *VGLL3* gene (Gat-Yablonski et al., 2011). The patient presented a face dysmorphic development suggesting alteration in the NC cell formation/migration. Curiously, *VGLL3* gene was also found to be significantly higher in human cartilage presenting endemic osteoarthritis, suggesting its implication in cartilage development (Wang et al., 2009). Our results emphasize the role of *vgll3* in the genetic regulatory network that controls cell-cell and cell-matrix interactions that could explain its essential function in CNC migration.

### Ets1 is a new partner of vgll3

We have shown that vgll3 can interact in the embryo with tead1 or tead2 as expected (Chen et al., 2004; Kitagawa, 2007). However, we found that the complex vgll3/tead2, unlike vgll3/tead1, is unable to induce *pax3, zic1*, *snail2 or N-cadherin* expression in animal cap cells. This suggests that the protein complexes vgll3/tead1 and vgll3/tead2 have distinct cis-regulatory targets or that animal cap cells are missing factors, present in the embryo that are required for *pax3* induction by vgll3/tead2. Alternatively, this might be reminiscent to what has been observed in *Drosophila* where the binding of Vestigial to Scalloped can switch the DNA-binding selectivity of Scalloped (Halder and Carroll, 2001).

Our results establish that tead1 is not the only transcription factor that conveys vgll3 activity *in vivo.* Indeed, vgll3 and ets1 can interact in the embryo and, when co-expressed, can stimulate a MCAT-luciferase reporter gene. Therefore, it is conceivable that vgll3 can bind either to tead or ets1 depending on both cell context and relative affinity of partners. A recent report has shown that ets1 represses NC formation through downregulation of BMP signaling (Wang et al., 2015). Whether this effect is modulated by vgll3 is unknown but it may be noted that gain- or loss-of-function of vgll3 and ets1 give the same phenotype with regard to trigeminal nerve formation, NC migration and defects in its derivatives (this work and Wang et al., 2015). Vgll3 as a new partner of ets1 was unexpected and is very challenging as ets1 is also a proto-oncogene and VGLL3 has been proposed to play a role in tumor progression (Antonescu et al., 2011; Cody et al., 2007, 2009; Gambaro et al., 2013; Hallor et al., 2009; Helias-Rodzewicz et al., 2010). In the future, it will be interesting to determine the relative affinity of vgll3 for tead and ets1 and the repertoire of target genes for the two complexes. Finally, we have evidence that the conserved histidine repeat in vgll3 protein is required for its transcriptional activity suggesting that this region is part of the transcriptional activation domain.

**Fig. 9.**
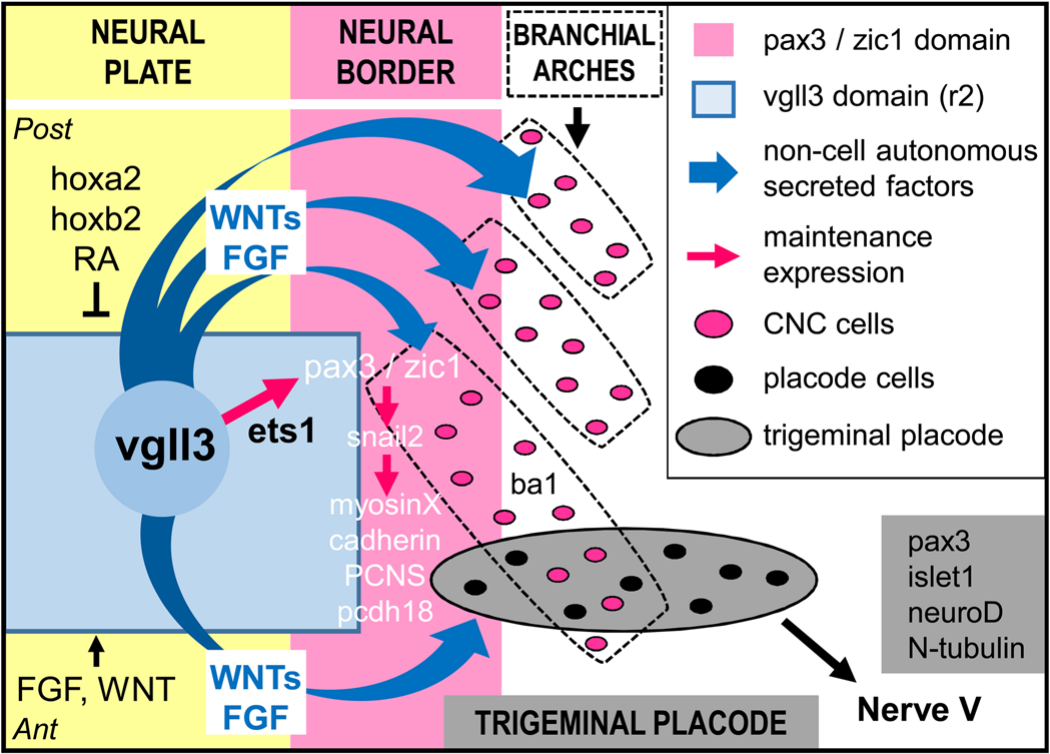
**Proposed model showing vgll3 function in trigeminal placode/nerve formation and CNC migration.** Vgll3 acts via secreted molecules such as WNT or FGF on neural border-expressed genes to control the formation of branchial arches and trigeminal placode. Vgll3 can interact with ets1 and sustain *pax3/zic1* expression and their downstream target genes. Ant, anterior; ba1, branchial arch1; CNC, cranial neural crest; Post, posterior; RA, retinoic acid.

In summary, our results provide the first evidence of the function of vgll3 during vertebrate development. Clearly, vgll3 is critical for trigeminal placode and nerve formation. Moreover, although vgll3 does not play a direct role in NC formation, it is required for their migration. We propose that vgll3 fulfill all these properties mainly through the activation of both wnt and FGF signals (Fig. 9). One major finding of our work is that ets1 is a novel partner of vgll3. This suggests that vgll3 can regulate distinct gene targets and activate or repress signaling pathways depending on its association with different transcription factors. This should be helpful in our exploration of its function in mammalian cells and for scientific community to provide new target genes for vestigial-like members associated with the new transcription factor, ets1.

## MATERIALS AND METHODS

### Ethics statement

This study was carried out in accordance with the European Community Guide for Care and Use of Laboratory Animals and approved by the Comité d’éthique en expérimentation de Bordeaux (No. 33011005-A).

### Plasmids and probes

Plasmid containing cDNAs encoding *X. laevis* vgll2 (IMAGE clone 4930090, accession number BC056001) and ets1 (IMAGE clone 8549297, NM_001087613) were obtained from Geneservice and Source BioScience, respectively. cDNA encoding *Xenopus laevis* vgll3 (XL405a05ex, accession number BP689606) was obtained from the National BioResource Project (www.nbrp.jp). The 5′-sequence of *vgll3* mRNA was obtained by 5′-RACE (Invitrogen). Coding sequences for *tead1*, *tead2* and *ets1* were subcloned in pCS2+MT vector. Cloning strategies for *HA-vgll3* cDNAs are indicated in Table S1.

### Embryo and explant manipulation

*Xenopus laevis* embryos were obtained and staged using current protocols (Nieuwkoop and Faber, 1975; Sive et al., 2000). All mRNAs were synthesized using the Message Machine kit (Ambion, Foster City, USA) and injected at the following doses: *noggin* (500 pg), *FGF8,wnt8a* (100 pg), *pax3GR*/*zic1GR* (100 pg each), *hoxa2* (70 pg), *hoxb2*, *tead1*, *tead2* (50 pg), *vgll3* (0.25-1 ng), *vgll3mis* (0.5 ng) and *vgll3Δhis*, *vgll2*, *ets1* (1 ng). For retinoic acid (RA) (Sigma-Aldrich) treatment, embryos were treated at stage 8 with 10^−6^ M to 10^−8^ M or with DMSO for control. Pax3GR- and zic1GR-injected embryos were cultured in 0.1× MMR with or without 10 µM dexamethasone from stage 10.5. Silencing of selected genes was performed using antisense morpholino oligonucleotides (GeneTools) (Table S2).

Synthetic mRNAs or MOs were co-injected with 250 pg *β-galactosidase* (lacZ staining) or *gfp* mRNA as a lineage tracer. Animal caps were dissected from early stage 9 embryos and cultured until appropriate stages before RNA extraction and RT-PCR analysis (Naye et al., 2007; Tréguer et al., 2009). Primers are listed in Table S3. All results shown are representative of three independent experiments.

### Whole-mount ISH and immunostaining

Whole-mount ISH was carried out using a standard protocol (Harland, 1991). For double ISH, probes were labelled with DIG and fluorescein or both with DIG. Staining was performed with Fast Red and BM-Purple or both with BM-Purple. Immunostaining was performed following standard procedures (Sive et al., 2000). Antibodies are described in Table S4. Embryos were embedded in agarose before sectioning.

### Immunoblotting

Embryos were lysed in RIPA buffer (PBS, 1% triton, 1% NP40, 0.05% SDS, 1 mM PMSF and proteinase inhibitors) (Roche, Boulogne-Billancourt, France). Proteins extracted from the equivalent of one embryo were loaded on 12% SDS-PAGE and transferred on nitrocellulose membranes. Proteins were reacted with the antibodies (Table S4) and staining was visualized using the enhanced chemiluminescence detection kit (GE Healthcare, Velizy-Villacoublay, France).

### *In vitro* translation

*In vitro* transcribed mRNAs (0.5 µg) were translated in lysate reticulocytes (Promega, Charbonnieres les bains, France) according to the manufacturer’s instructions and in the presence of 100-200 ng of MOs and [^35^S] methionine. The reaction products were analyzed by 12% SDS-PAGE followed by autoradiography.

### Alcian Blue staining

Stage 47 embryos were fixed in MEMFA and stained in 0.05% Alcian Blue/30% acetic acid in ethanol. Embryos were washed through a glycerol series before manual cartilage dissection. Cartilages were embedded in paraplast for serial sections.

### Migration assay

Migration assay was performed from CNC explants as described before (Borchers et al., 2000; Alfandari et al., 2003). CNC explants from GFP-labeled embryos were grafted homotypically into unlabeled host embryos (*in vivo*) or plated on bovine plasma fibronectin (*in vitro*) (10 µg/ml, Sigma-Aldrich). The ratio of spreading of the explants was measured by comparing the relative surface area at 18 h of culture to that at 3 h. The surface area of individual CNC explants was performed using the Image J plugin (http://rsb.info.nih.gov/ij/features.html). Student's *t*-test was performed to determine significant effects of *vgll3* mRNA (*n*=15) compared to *gfp* mRNA (*n*=19) injections, and the effect of v3MO (*n*=24) compared to cMO (*n*=12) injections.

### TUNEL

TUNEL assay was completed using a protocol previously described (Hensey and Gautier, 1997).

### Immunoprecipitation

Batches of 30 embryos injected with relevant mRNAs were lysed at gastrula stage in 50 mM Tris-HCl (pH 7.5), 150 mM NaCl, 0.5% Nonidet P-40, 1 mM PMSF and proteinase inhibitors (Roche). Pre-cleared proteins were incubated with appropriate antibodies (2 µg) (Table S4) and then incubated with protein A sepharose beads (Sigma-Aldrich). Bead pellets were boiled in SDS sample buffer before loading onto 10% SDS-PAGE gels. Bound antibodies (anti-myc or anti-HA) were detected with HRP-conjugated EasyBlot anti-mouse IgG (diluted at 1/1000) (GeneTex, Wembley, UK) and visualized as before.

### Cell transfection and reporter gene analysis

HEK293 cells were seeded at 6×10^4^ cells/cm^2^ and co-transfected with a TK-driven renilla construct (pRL-TK, Promega) for normalization of transfection efficiency, together with the pGL284LUC construct (Pasquet et al., 2006), or the pGL284LUC construct in addition to DNA constructs expressing tead1, ets1, vgll3 or vgll3ΔTDU (500 ng/well). Transfection assay was performed using X-treme gene (Roche) according to the manufacturer's instructions. Luciferase activity (Dual Luciferase, Promega) was quantified with a Varioskan Flash (Thermo Fisher Scientific) and results were calculated from duplicate samples of three independent repeats.

### Statistical analysis

Quantitative data are presented as mean±s.e.m. and were analyzed using Student's unpaired two-tailed test. Statistical significance was defined at *P*<0.05.
